# Identification and characterization of the first endogenous phospholipase A_2_ inhibitor from a non-venomous tropical snake, *Boa constrictor* (Serpentes: Boidae)

**DOI:** 10.1590/1678-9199-JVATITD-2019-0044

**Published:** 2020-03-13

**Authors:** Consuelo L. Fortes-Dias, Diego Henrique Fagundes Macedo, Rafaella Pereira Barbosa, Gabriel Souza-Silva, Paula Ladeira Ortolani

**Affiliations:** 1 Research & Development Center, Ezequiel Dias Foundation (FUNED), Belo Horizonte, MG, Brazil.

**Keywords:** Phospholipase A_2_ inhibitor, Phospholipase A_2_, Boidae, Snakes, Rattlesnake venom

## Abstract

**Background:**

Endogenous phospholipase A_2_ inhibitors from snake blood (sbPLIs) have been isolated from several species around the world, with the primary function of self-protection against the action of toxic phospholipases A_2._ In American snakes, sbPLIs were solely described in pit vipers, in which the natural protection role is justified. In this study, we described a sbPLI in *Boa constrictor* (popularly known as *jiboia*), a non-venomous snake species from America.

**Methods:**

PLA_2_ inhibitory activity was tested in the blood plasma of *B. constrictor* using *C. d. terrificus* venom as the enzyme source. Antibodies developed against CNF, a sbγPLI from *Crotalus durissus terrificus,* were used to investigate the presence of homologues in the blood plasma of *B. constrictor*. A CNF-like molecule with a PLA_2_ inhibitory activity was purified by column chromatography. The encoding gene for the inhibitor was cloned from *B. constrictor* liver tissue. The DNA fragment was cloned, purified and sequenced. The deduced primary sequence of interest was aligned with known sbγPLIs from the literature.

**Results:**

The blood plasma of *B. constrictor* displayed PLA_2_ inhibitory activity. A CNF-like molecule (named BcNF) was identified and purified from the blood plasma of *B. constrictor*. Basic properties such as molecular mass, composing amino acids, and pI were comparable, but BcNF displayed reduced specific activity in PLA_2_ inhibition. BcNF showed highest identity scores (ISs) with sbγPLIs from pit vipers from Latin America (90-100%), followed by gamma inhibitors from Asian viperid (80-90%). ISs below 70% were obtained for BcNF and non-venomous species from Asia.

**Conclusion:**

A functional sbγPLI (BcNF) was described in the blood plasma of *B. constrictor.* BcNF displayed higher primary identity with sbγPLIs from Viperidae than to sbγPLIs from non-venomous species from Asia. The physiological role played by sbγPLIs in non-venomous snake species remains to be understood. Further investigation is needed.

## Background

Secretory phospholipases A_2_ are widely distributed as toxic components of snake venoms. A number of snake species express endogenous snake blood phospholipase A_2_ inhibitors (sbPLIs). This kind of molecules was first described in venomous snakes with the primary function of self-protection against an eventual presence of snake venom PLA_2_ (svPLA_2_) in their own blood stream [1, 2]. According to the presence of known domains from mammal proteins - C-type lectin-like, tandem leucine-rich repeats (LRRs), or three-finger motifs - sbPLIs were grouped into alpha (α), beta (β) or gamma (γ) structural classes, respectively [3]. Comparable inhibitors were later identified in a number of non-venomous species [4, 5, 6, 7, 8, 9]. Whether venomous or not, some snake species express sbPLIs belonging to up to three different structural classes simultaneously [3, 7, 10, 11]. 

SbγPLIs are the most widely distributed inhibitors among elapid and viperid species from the Old and New World [12, 13, 14]. Concerning non-venomous snakes, as far as we know, until now sbγPLIs were solely purified from Asian species [4, 5, 6, 7, 8, 9]. With that in mind, we investigated the presence of this kind of inhibitor in *Boa constrictor* - a non-venomous tropical snake - popularly known as *jiboia*. We identified a functional sbγPLI, cloned the encoding gene from liver tissue and structurally characterized the deduced protein. The sbγPLI was named BcNF by analogy with CNF (*Crotalus* neutralizing factor), a prototype of this class of inhibitors previously isolated from the South American rattlesnake, *Crotalus durissus terrificus* [15, 16].

## Methods

### *Boa constrictor* blood plasma and liver tissue collection

Heparinized blood plasma and liver tissue fragments were collected from a *Boa constrictor* specimen captured in the municipality of Contagem (19º55'54" S, 44º03'13" W), in the Brazilian state of Minas Gerais. The specimen was kept in captivity in the Serpentarium of Ezequiel Dias Foundation until death by natural causes. The whole blood was collected immediately after the animal death, centrifuged for plasma separation and clarified using a 0.22-µm microfilter. The total protein content was estimated by spectrophotometry readings at 280 nm. One optical density unit was considered to be equivalent to 1 mg/mL of protein. Liver fragments were collected in DEPC-treated tubes and quickly frozen in liquid nitrogen. Whenever applicable, blood plasma and tissue liver from *C. d. terrificus* specimens were used as reference.

### Fractionation of *B. constrictor* blood plasma

Five hundred microliters of *B. constrictor* blood plasma were diluted to 10 mL with 25 mM Tris-HCl, 0.1 M NaCl pH 8.7 (buffer A) and dialyzed against the same buffer to ensure ionic equilibrium. After centrifugation to remove any insoluble material, the supernatant was loaded into an anion exchange column (Hitrap QFF 1mL, GE HealthCare). Protein elution was performed with a linear gradient of 25 mM Tris-HCl, pH 8.7, containing 2.0 M NaCl (buffer B), under a flow rate of 1 mL/min. Fractions with inhibitory activity (1 mL each) were pooled, 4-fold diluted with a saturated ammonium sulfate (SAS) solution and loaded into hydrophobic interaction columns connected in series [four columns HiTrap Phenyl FF 5 mL (low sub) column, GE HealthCare]. Elution was performed with a decreasing salt gradient under a flow of 5 mL/min. Total protein concentration was estimated by optical density readings of the eluted fractions at 280 nm. 

### Inhibition of PLA_2_ activity

The crude venom of *C. d. terrificus* was used as a source of PLA_2_. Increasing volumes of snake blood plasma with known protein concentration were preincubated with a fixed concentration (50 μg/mL) of *C. d. terrificus* venom for 30 min at 37°C. The same procedure was applied to purified fractions, after dialysis against 25 mM ammonium formats, pH 6.5, whenever necessary. Residual PLA_2_ activity was evaluated by measuring the clearing halos (in mm) of hydrolysis in agar gels with incorporated hen egg yolk suspension [17]. Negative (PBS) and positive (no blood plasma) controls were run in parallel. Inhibition curves were constructed by plotting the halo diameter against protein concentration in logarithm scale. Data were analyzed by linear regression using least squares method in the Graph Prism 6.0 for Mac OS X (GraphPad software Inc., California). Curve limits were calculated with 95% of confidence level. Specific activities were represented by curve slopes and expressed by mean ± S.D. Whenever applicable, regression line slopes were statistically compared in pairs. 

### SDS-PAGE and western blotting

*B. constrictor* blood plasma and purified BcNF were analyzed by SDS-PAGE in a 15% homogeneous or in an 8-25% gradient Phast® gel (Phast System®, GE HealthCare). Western blotting was revealed with rabbit anti-CNF IgG (0.5 mg/mL), followed by commercial anti-rabbit IgG-peroxidase antibody (A0545, Sigma) at a 1:5000 dilution. The color reaction was developed with DAB (3,3' diaminobenzidine tetrahydrochloride) in the presence of H_2_O_2_. 

### RNA extraction and cDNA synthesis

Total RNA was isolated from about 50 mg of *B. constrictor* liver tissue using Trizol® (Invitrogen, USA) following the manufacturer’s instructions. RNA integrity was analyzed by gel electrophoresis in a 0.8% agarose gel using TBE (89 mM Tris base, 89 mM boric acid, 2 mM EDTA, pH 8.0) as running buffer. RNA bands were visualized under UV light, after staining with ethidium bromide. After cDNA synthesis using 2 to 5 (g of total RNA and oligo(dT)12-18 primer (First-Strand Synthesis kit, Invitrogen, USA), polymerase chain reactions were carried out with specific oligonucleotides based on the primary structure of CNF [15]: 3’CGCTCATGTGACTTTTGTCAC5’ (sense, amino-terminus), 3’TCAGAGGCTTGCCAATCTGATG5’ (antisense, carboxy-terminus). A housekeeping gene (β?actin) was PCR-amplified in parallel, in the presence of adequate oligonucleotides. 

Fresh PCR products were cloned into the pGEM-T vector (Promega, USA) following the manufacturer’s instructions. Insert-containing clones were isolated after PCR screening of transformed NM522 *E. coli.* Negative control contained no DNA. Amplified products were analyzed by electrophoresis in 1.0 % agarose gels in TBE buffer, in the presence of ethidium bromide. DNA from three positive clones were completely sequenced by the dideoxy chain termination method [18] on an automated ABI Prism 310 Genetic Analyzer (Perkin Elmer Applied Biosystems, USA) with the Big Dye Terminator Cycle Sequencing Ready Reaction (Perkin Elmer Applied Biosystems, USA). M13 forward and M13 reverse oligonucleotides were used as primers.

The cycling conditions were 3 min at 94°C, 35 cycles of 30 sec at 94°C, 30 sec at 55°C and 1 min at 72°C, followed by an extension period of 5 min at 72°C in a TC412 thermocycler (Techne).

### Primary/secondary structure predictions and multiple alignment

Three complete reads in both directions were assembled and aligned against each other. The consensus sequence was used to deduce the primary structure and main basic properties of BcNF. The secondary structure was predicted using the Chou Fasman algorithm. Multiple sequencing alignments with primary structures of other sbγPLIs were performed using the ClustalW algorithm and a Gonnet’s similarity matrix was subsequently generated. Inclusion criterium for sbγPLIs was the access to chemically determined or deduced primary structures in public data bases. For species with two or more sequence deposits due to isoforms, calculated consensus was taken as representative of the inhibitor. Signal peptides were removed, whenever necessary. The sbγPLIs from the following snake species were aligned: *Bothrops alternatus* (ABV91326/7), *Bothrops erythromelas* (ABV91328/9), *Bothrops jararaca* (ABV91330/1), *Bothrops jararacussu* (ABV91332/3), *Bothrops moojeni* (ABV91332/5), *Bothrops neuwiedi* (ABV91336/7), *Crotalus durissus terrificus* (AAA19162), *Elaphe climacophora* (BAH47550), *Elaphe quadrivirgata* (BAA83078), *Gloydius brevicaudus* (formerly *Agkistrodon blomhofii siniticus*) (BAA86970), *Lachesis muta* (AAR04437/8), *Malayopython reticulatus* (formerly *Python reticulatus*) (AAF73945), *Notechis scutatus* (CAB56615/6/7), *Oxyuranus microlepidotus* (AAF23784), *Oxyuranus scutellatus* (AAF23781), *Protobothrops flavoviridis* (formerly *Trimeresurus flavoviridis*) (BAA24502)*, Protobothrops elegans* (BAJ14719/20/21), *Pseudonaja textilis* (AAF23783), *Sinonatrix annularis* (JN975878)*.* All the procedures were performed using the MacVector 16.0.10 software (Mac Vector Inc., USA) with default parameters. 

## Results

### Identification and purification of BcNF from *B. constrictor* blood plasma

**Figure 1:**
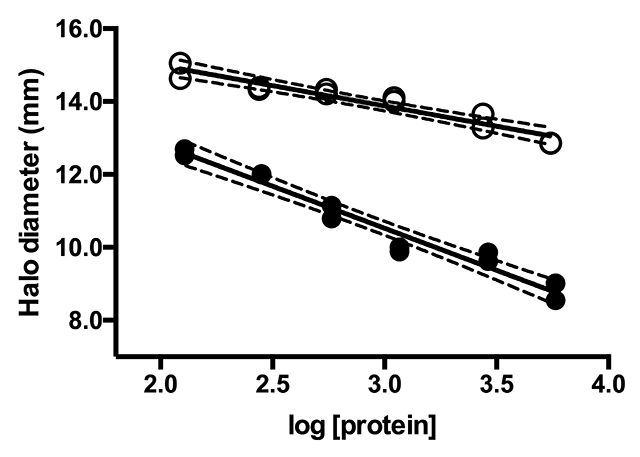
Inhibition curves of PLA_2_ activity of *C. d. terrificus* venom by the blood plasma of *Boa constrictor* (white dots). The blood plasma of *C. d. terrificus* was used as reference (black dots). Curve equations: y = (-1.112 ± 0.1075)x + (17.22 ± 0.3193) for *B. constrictor* and y = (-2.307 ± 0.1498)x + (17.45 ± 0.4478) for *C. d. terrificus*, with determination coefficient (R^2^) of 0.9145 and 0.9546, respectively. The 95% confidence intervals of the best fit curves are indicated by dashed lines.

First, the blood plasma of *B. constrictor* was tested for inhibition of *C. d. terrificus* venom PLA_2_ (Fig. 1). Inhibition was observed, although to a lesser extent when compared to *C. d. terrificus* blood plasma (positive control). Specific activities for PLA_2_ inhibition were: -1.112 ± 0.1075 and -2.307 ± 0.1498 for *B. constrictor* and *C. d. terrificus* blood plasma, respectively. These activities were statistically different (*p* < 0.0001). PLA_2_ inhibition activity was significantly lower for *B. constrictor* blood plasma.

**Figure 2:**
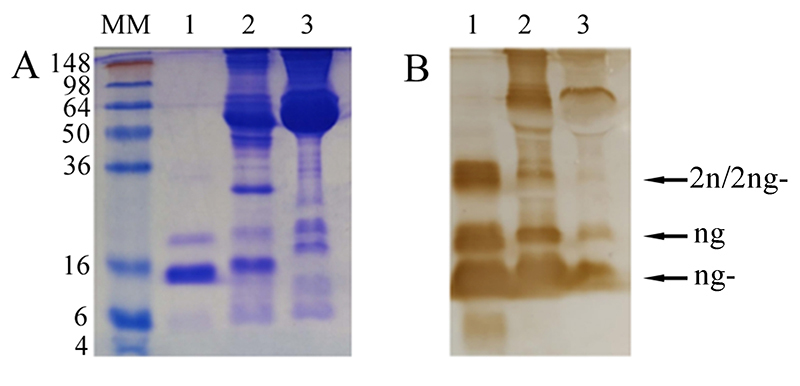
(A) SDS-PAGE 15% after staining with Coomassie Blue and (B) Western blotting revealed with anti-CNF IgG. Lanes: MM - molecular marker (in kDa) (SeeBlue Plus 2 Pre-stained Protein Standard, Invitrogen); 1 - CNF (20 µg); 2 - *C. d. terrificus* blood plasma (80 µg); 3 - *B. constrictor* blood plasma (80 µg). The arrows indicate non-glycosylated monomer (ng-), glycosylated monomer (ng) and possible dimers (2ng/2ng-).

Following, we investigated whether the observed inhibition could be due to the presence of a sbγPLIs. Western blotting revealed the presence of a CNF-like molecule in the blood plasma of *B. constrictor*. Two main protein bands were recognized by anti-CNF antibodies (Fig. 2), with apparent molecular masses roughly corresponding to glycosylated (ng) and non-glycosylated (ng-) monomers. A fainter band was present with mol. mass of possible dimers (2ng/2ng-). The result indicated the presence of a sbγPLIs, named BcNF, in the blood plasma of *B. constrictor*.

**Figure 3:**
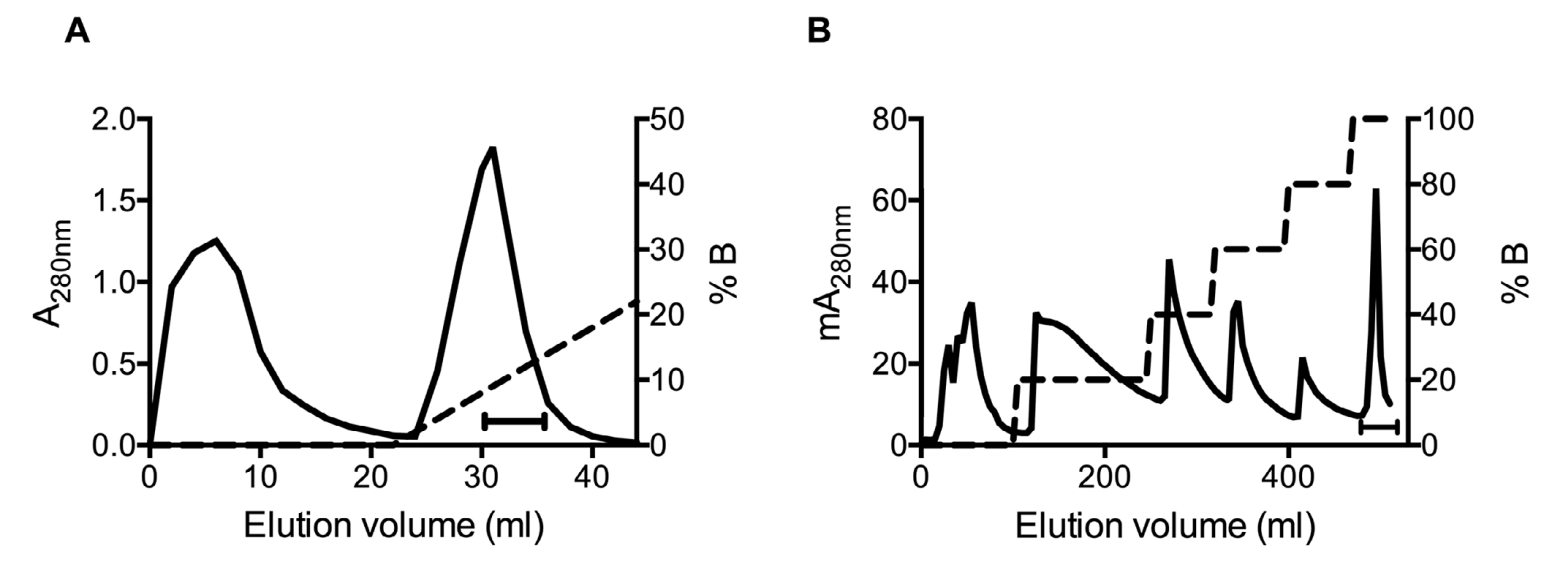
Purification of BcNF from *B. constrictor* blood plasma. **(A)** Anion-exchange and **(B)** hydrophobic interaction chromatograms. Elution gradients are indicated by dotted lines. PLA_2_ inhibitor-containing fractions are indicated by horizontal bars.

**Figure 4:**
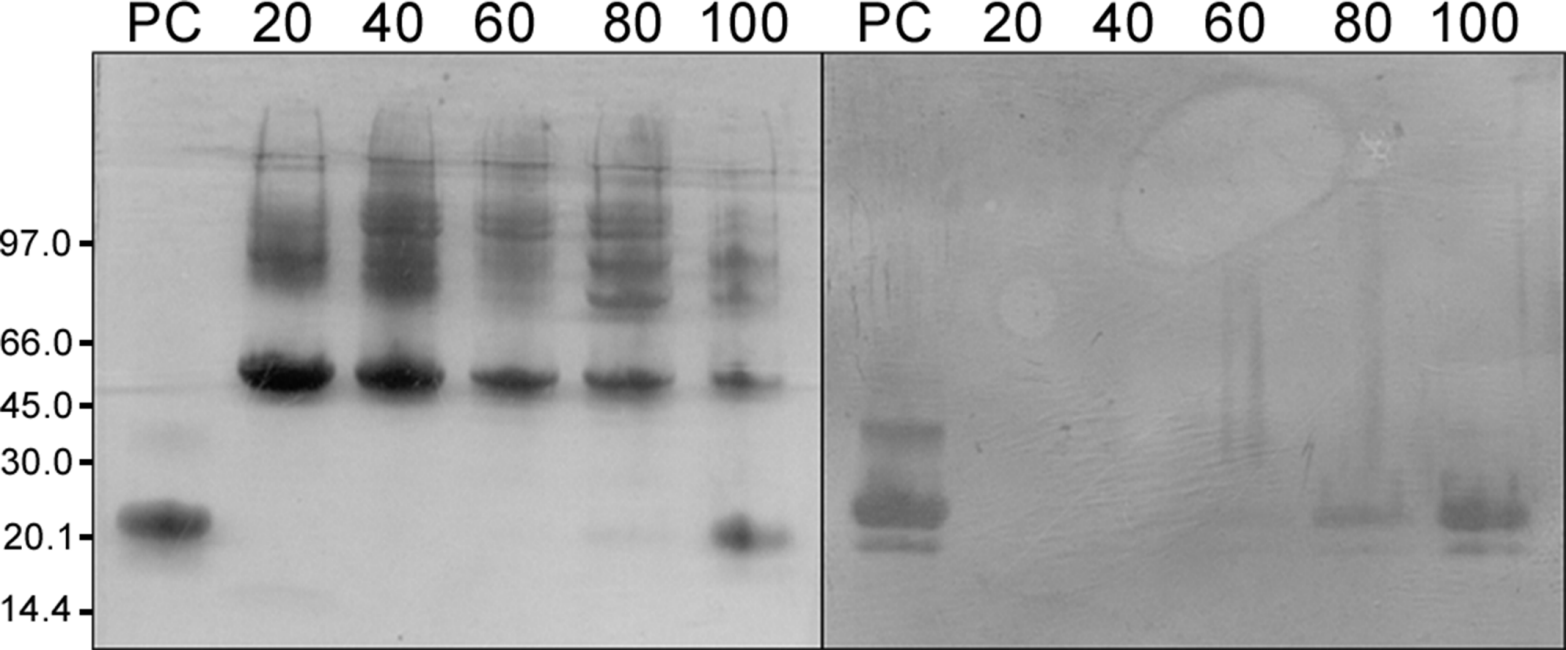
(A) SDS-PAGE in 8-25% gel after silver staining and (B) Western blotting developed with anti-CNF IgG. PC: positive control (CNF). Lanes are numbered on top according to percentages of eluent B (ultrapure water) in the hydrophobic interaction chromatography.

**Figure 5:**
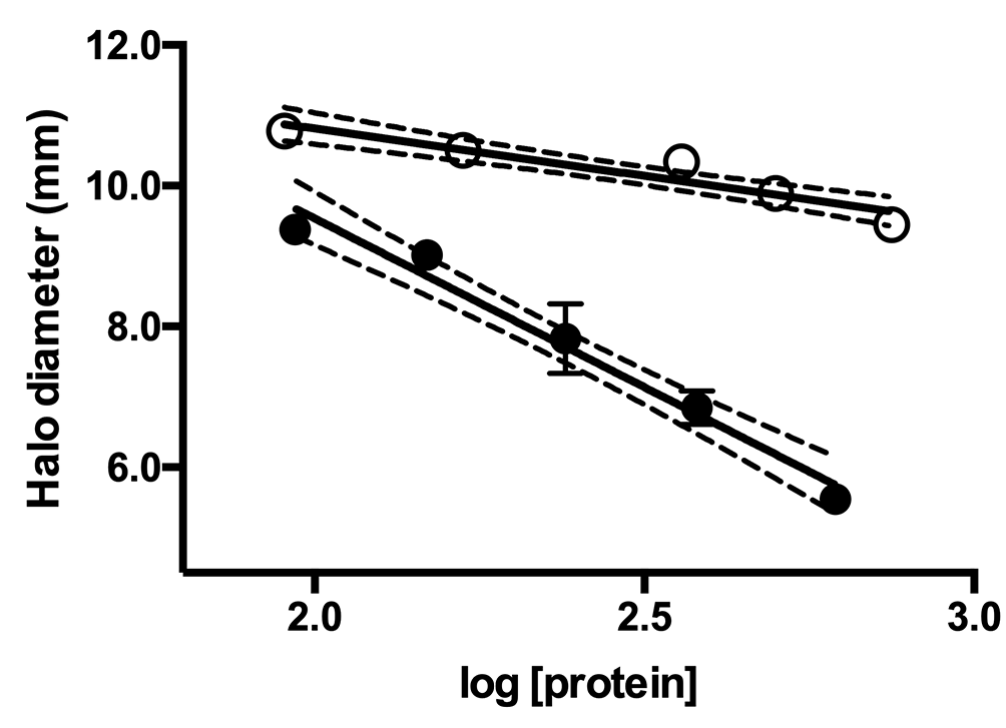
Inhibition curves of PLA_2_ activity of *C. d. terrificus* venom by BcNF isolated from *B. constrictor* blood plasma (white dots). CNF from *C. d. terrificus* snakes was used as positive control for PLA_2_ inhibition (black dots). Curve equations: y = (-1.344 ± 0.1705)x + (13.50 ± 0.4235) for BcNF, and y = (-4.797 ± 0.3434)x + (19.13 ± 0.4478) for CNF, with determination coefficient (R^2^) of 0.8860 and 0.9606, respectively. The 95% confidence intervals of the best fit curves are indicated by dashed lines.

BcNF was isolated from *B. constrictor* blood plasma using two chromatographic steps: an ionic exchange followed by a hydrophobic interaction. The eluted fractions were assayed for PLA_2_ inhibition (Fig. 3). Fractions from the second purification step presenting inhibitory activity were combined and submitted to electrophoresis and Western blotting using anti-CNF IgG. A CNF-like molecule (BcNF) was mostly eluted with 100% of ultrapure water (Fig. 4). BcNF and CNF (positive control) at varying concentrations were assayed for PLA_2_ inhibition (Fig. 5). Calculated specific activities were -1.344 ± 0.1705 and -4.797 ± 0.3434 for BcNF and CNF, respectively. These activities were statistically different (*p* < 0.0001). BcNF inhibited PLA_2_ at a significant lesser extent compared to CNF.

### BcNF cloning from *B. constrictor* liver tissue

**Figure 6:**
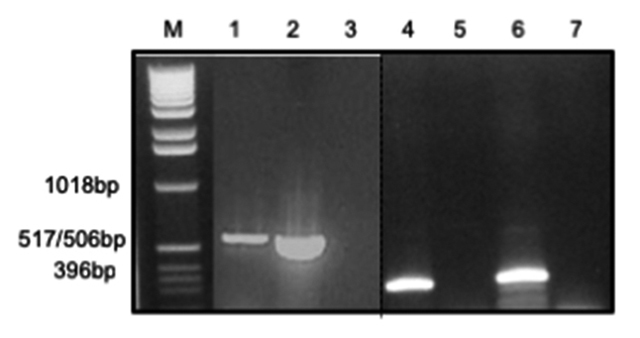
Electrophoresis of RT-PCR products after amplification of liver tissue with specific primers for CNF (left side) or β-actin (right side). M: molecular marker 1 kb DNA ladder (Gibco-BRL). Lanes 1 and 4: *B. constrictor* liver; lanes 2 and 6: *C. d. terrificus* liver (reference); lanes 3 and 7: negative control (no DNA); lane 5: no reverse transcriptase in the reaction.

The integrity of extracted RNA from *B. constrictor* liver tissue was confirmed by the unique presence of characteristic bands corresponding to 18S and 28S ribosomal RNAs (data not shown). After RT-PCR in the presence of specific primers for CNF, an amplicon of about 545 bp confirmed the encoding of a CNF-like protein in the liver tissue of *B. constrictor* (Fig. 6). The DNA fragment was cloned, purified and sequenced for further analysis.

### Deduced primary structure and chemical properties predictions of BcNF

**Figure 7:**
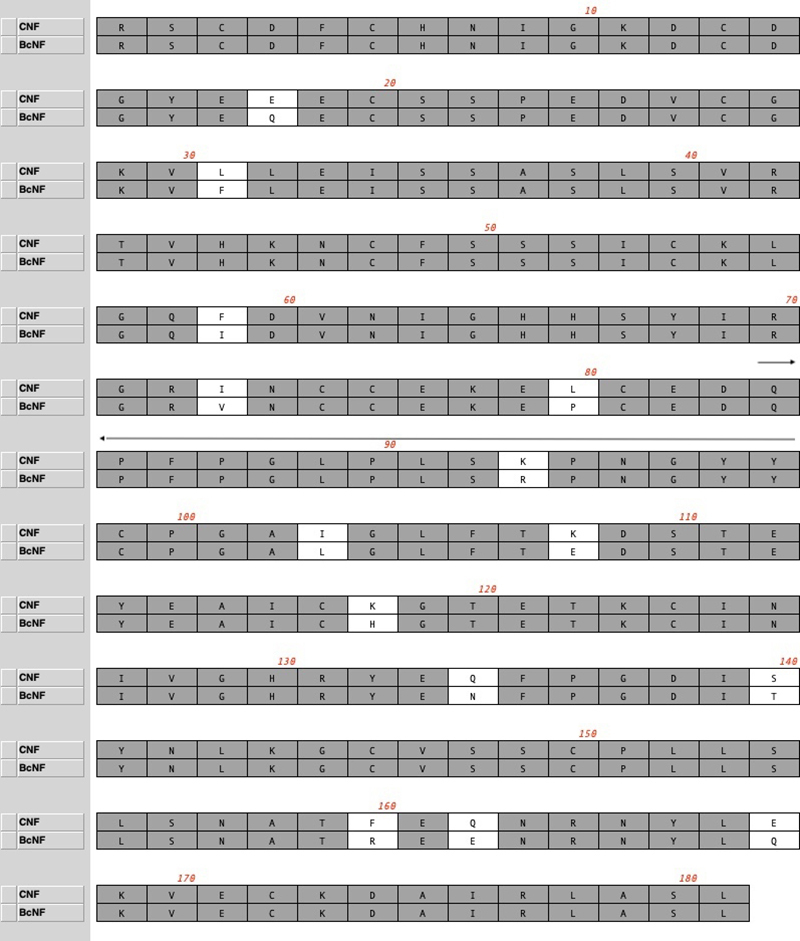
Alignment of the deduced primary structure of BcNF (sbγPLI from *B. constrictor*) and CNF (sbγPLI from *C. d. terrificus*). Identical amino acids are in grey background. Amino acid substitutions are in white background. The decapentapeptide Q^84^PFPGLPLSRPNGYY^98^ is indicated by a continuous black arrow above the numbering line.

The deduced primary sequence of mature BcNF was compared to that of CNF. Both proteins are composed of 181 amino acids, including 16 conserved cysteines and a single putative N-linked carbohydrate site at Asn^157^. Fourteen amino acid substitutions were noted in BcNF when compared to CNF, one of them (R^93^/K^93^) within a segment proposed before for sbγPLIs interaction with PLA_2_ (Fig. 7). Basic properties of BcNF and CNF are summarized in Table 1. 

**Table 1:** Comparison of basic properties of BcNF (sbγPLI from *B. constrictor*) and CNF (sbγPLI from *C. d. terrificus*)

Property	BcNF	CNF
Molecular mass (Da)	20074.57	20058.69
Isoelectric point (pI)	5.51	5.55
Composing amino acids		
Total (no.)	181	181
Chemical character (%)		
Non-polar	30.4	30.9
Polar	43.0	42.4
Acidic	13.2	13.3
Basic	13.3	13.3

**Figure 8:**
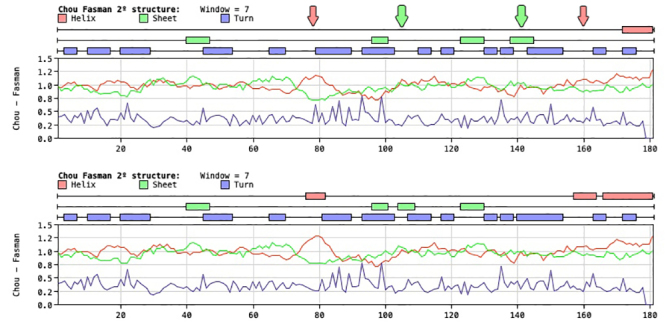
Secondary structure predicted for BcNF (top) compared to CNF (bottom). The differences are indicated by arrows on top of BcNF structure, using the same color as in the structural diagram.

Amino acid substitutions, in general, lead to a decrease in the number of α-helixes from three in CNF to one in BcNF, besides a displacement of beta sheets in the predicted secondary structures of the proteins (Fig. 8). 

### Multiple sequence alignment of BcNF and other sbγPLIs

**Figure 9:**
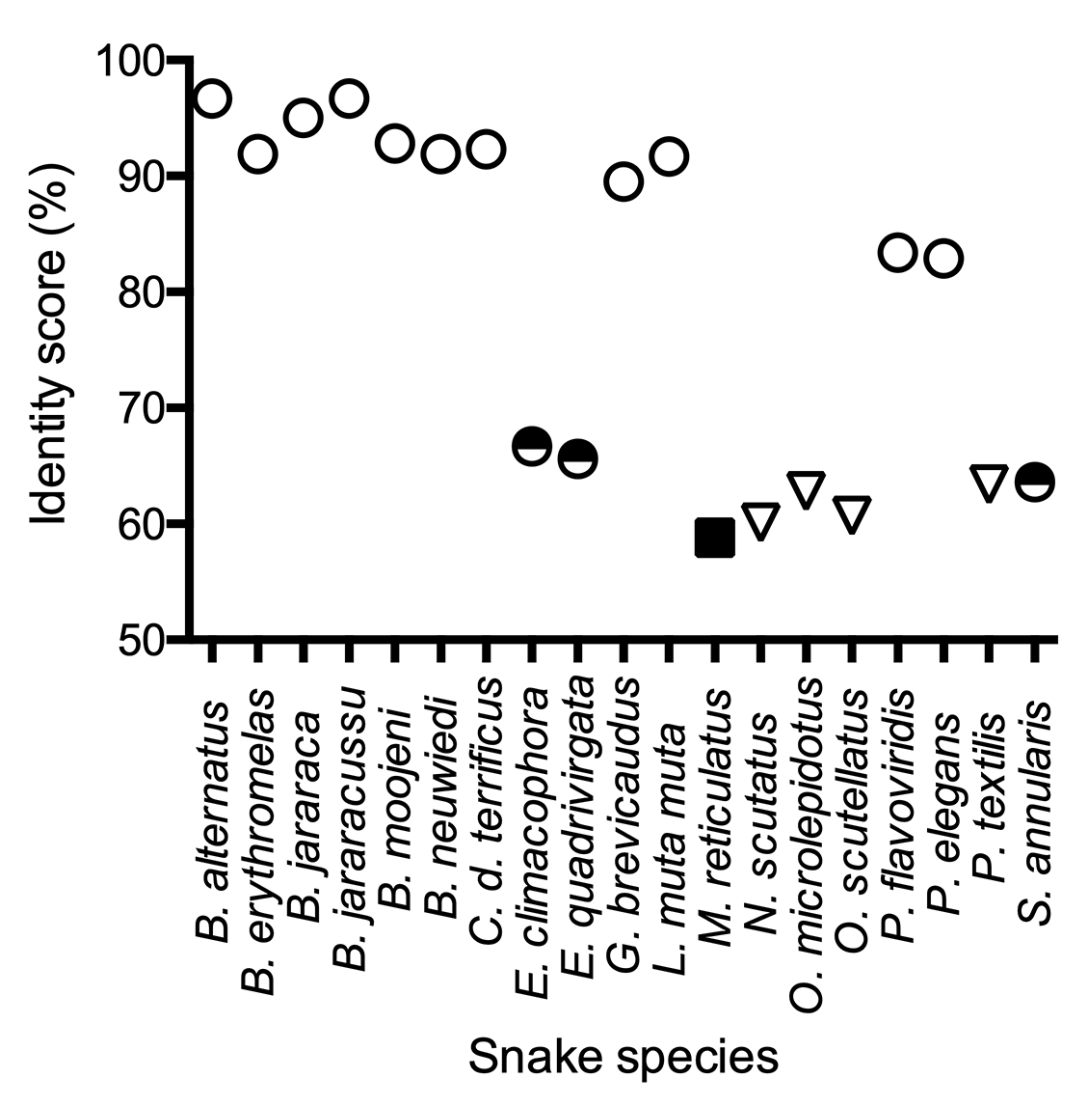
Graphical representation of the identity scores (ISs) obtained in Gonnet’s similarity matrix after multiple alignment of the deduced primary structure of BcNF with known sbγPLIs. Black/white circle: Colubridae, white triangle: Elapidae, white circle: Viperidae, black rectangle: Pythonidae.

The deduced primary sequence of BcNF was multiply aligned with sbγPLIs from venomous and nonvenomous snakes from Asia, Australia and Latin America (available as Additional file 1). A similarity matrix was generated (available as Additional file 2) and the identity scores (ISs) were graphically represented (Fig. 9). For BcNF and sbγPLIs from Latin American pit vipers, most ISs were within the last decile (90-100%). ISs above 80% were obtained for Asian viperid snakes. On the other hand, when BcNF was compared to sbγPLIs from non-venomous species from Asia, the ISs were below 70%. ISs below 70% were also obtained for Elapidae snakes. 

## Discussion

*Boa* is a Neotropical genus of snakes that occurs almost continuously from southern South America through to northern Mexico [19]. Historically recognized as monotypic, recent data based on the distinct morphological traits, color patterns exhibited by these snakes and the wide diversity of ecosystems they inhabit, collectively suggest that the genus contains multiple species [20]. In Brazil, *B. constrictor* (*sensu lato*) can be found all over the country, except in the extreme south [21]. It is an aglyphous species, devoid of venom or Duvernoy’s glands. Similarly to other henophidian snakes (boas, pythons and their kin), *B. constrictor* uses constriction to subdue and kill a wide range of prey - including lizards, birds and mammals - through an interesting modulated process mediated by the victim’s heartbeat [22]. Apparently, there is no need of an inhibitor for self-protection against toxic svPLA_2_. 

The detection of sbPLIs in non-venomous snake species is not a novelty. The first sbγPLIs was isolated from *E. quadrivirgata* [6]. The finding was later attributed to feeding habits of the species on venomous snakes [7]. However, another sbγPLI - named PIP for phospholipase A_2_ inhibitor from *Python* - was soon described in the non-venomous and non-ophiophagus species *Malayopython reticulatus* (formerly *Python reticulatus*) [8]. Since then, a number of sbγPLIs were detected in colubrid from Asia: *Dinodon rufozanatum* [5], *Elaphe carinata* [5], *E. climacophora* [7], *E. rufodorsata* [5], *E. teniura*, *Macropisthodon rudis* [9], *Synonatrix annularis* [4], and *Zaocys dhumnades* [5], in addition to xenodermatid *Achalinus rufescens* [5]. A structurally-related PIP homolog was also described in the non-venomous rock python (*P. sebae*) from Africa, although with poor PLA_2_ inhibition activity [23]. Regarding non-venomous snakes living in the American continent, studies are lacking on any sbPLI. 

*B. constrictor* inhibition of PLA_2_ was lower than that of *C. d terrificus* blood plasma. Similarly, BcNF was less active than CNF. Our results are in accordance with those described for *E. climacophora* and *E. quadrivirgata.* Respective sbγPLIs were detected at higher amounts in the former, and justified by the ophiophagous habits of the species [7]. It is important to note that, in addition to sbγPLIs, those Elaphe species express sbα? and sbβPLIs simultaneously in the circulating blood. We used antibodies developed against CNF to search for sbγPLI in *B. constrictor*. The detection of inhibitors from other structural classes is a possibility that cannot be discarded. 

BcNF is highly similar to CNF, with 14 substitutions in a total of 181 amino acids and an IS of about 90%. The molecular masses of the non-glycosylated monomers, calculated from amino acid compositions, are very close (Table 1). Band migrations in gel electrophoresis also indicated similar apparent molecular masses for monomers and oligomers (Fig. 7). Like CNF, BcNF is composed by a mixture of non-glycosylated (20 kDa) and glycosylated (22-24 kDa) monomers. For CNF, which is the main subject of study in our lab, the proportion between non-glycosylated and glycosylated varies according to the preparation. The sample loaded in SDS-PAGE (Fig. 2) was mostly non-glycosylated. However, it has been shown that the carbohydrate moiety is not essential for PLA_2_ inhibition by CNF [24]. The same might be true for BcNF. The tendency for oligomerization might be a shared property, too. In fact, the 16^th^, 113^th^, 132^nd^ and 166^th^ tyrosinyl residues, which were previously suggested to form the interface between monomers in the oligomerization of CNF, are maintained at the same positions in BcNF. These residues might be involved in the oligomerization of the latter also. BcNF was only tested against svPLA_2_ from *C. d. terrificus* venom, but the possibility of inhibition of other svPLA_2_ cannot be discarded. The decapentapeptide Q^84^PFPGLPLSRPNGYY^98^, which was previously proposed to be the best consensus motif possibly involved in the sbγPLIs interaction with PLA_2s_ is maintained in BcNF. The only amino acid replacement was conservative (R^93^/K^93^). 

Interestingly, BcNF appeared more closely related to sbγPLIs from Latin American pit vipers, and from Asian pit vipers to a lesser extent, than to those from non-venomous snakes from Asia described so far.

## Conclusion

A functional sbγPLI (BcNF) was described, for the first time, in the blood plasma of *B. constrictor,* a non-venomous species from America. BcNF displayed higher primary identity with sbγPLIs from pit vipers than with sbγPLIs from non-venomous species from Asia. Even with a growing number of sbγPLI identifications in the last years, the physiological role played by these proteins in non-venomous snake species remains to be clarified. 

### Abbreviations

IS: identity score; LRRs: tandem leucine-rich repeats; SAS: saturated ammonium sulfate; sbPLI: snake blood phospholipase A_2_ inhibitor; svPLA_2_: snake venom phospholipase A_2_.

## Supplementary material

The following online material is available for this article:

Additional file 1. Multiple alignment of sbγPLIs.

Additional file 2. Gonnet’s similarity matrix obtained after multiple sequence alignment of sbγPLI from data bases, except for *B. constrictor.*
