# Supplementary material for: Identification and characterization of the first endogenous phospholipase A2 inhibitor from a non-venomous tropical snake, Boa constrictor (Serpentes: Boidae)
Source: J Venom Anim Toxins Incl Trop Dis. 2020 Mar 13;26:e20190044. doi: 10.1590/1678-9199-JVATITD-2019-0044 (PMC7092641; doi:10.1590/1678-9199-JVATITD-2019-0044)
Supplement: Additional file 1. [file 1678-9199-jvatitd-26-e20190044-s1.pdf]

J Venom Anim Toxins incl Trop Dis, 2020, 26

Supplementary Material to "Identification and characterization of the first endogenous phospholipase A2 inhibitor from a non-venomous tropical snake, *Boa constrictor* (Serpentes: Boidae)"

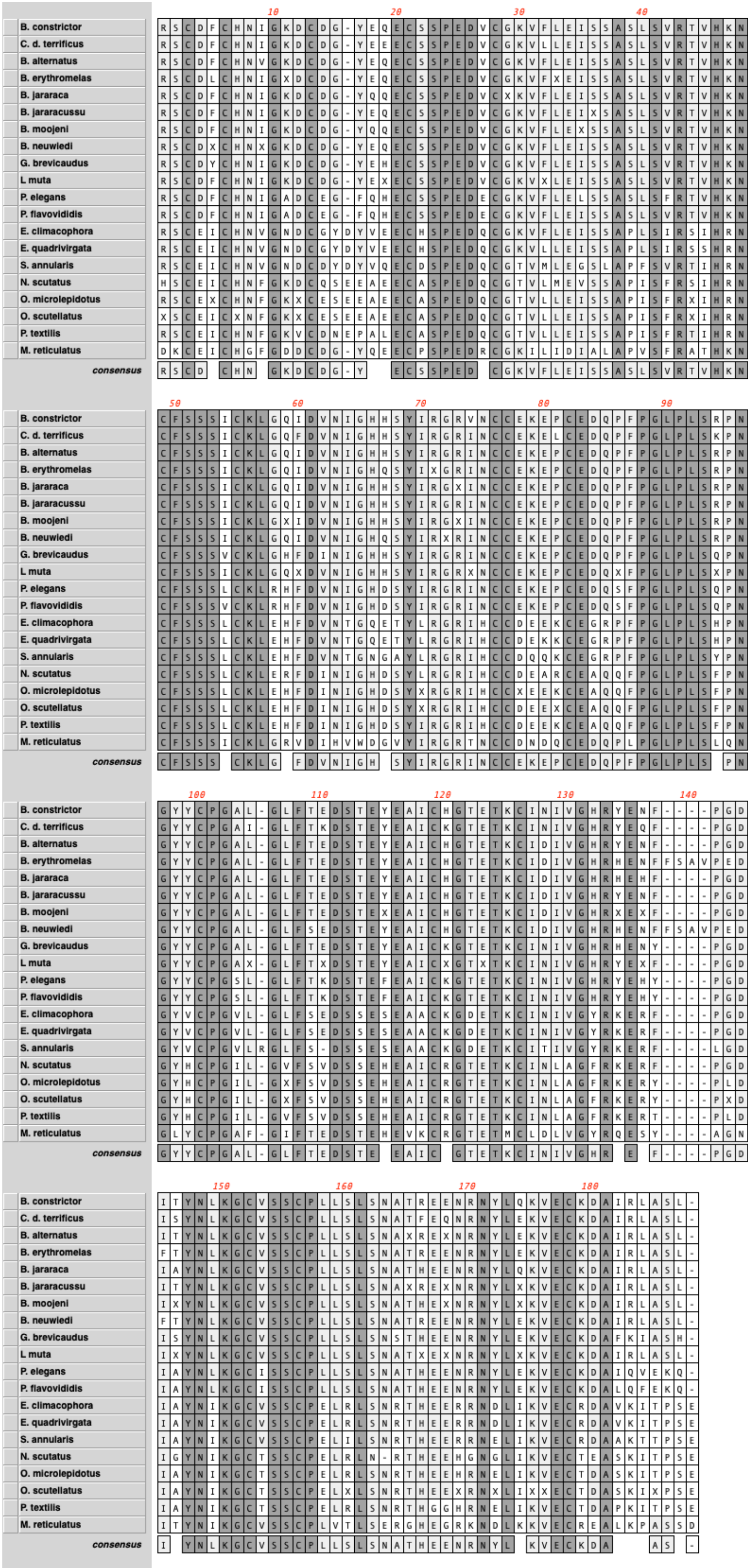

Additional file 1 - Multiple alignment of sbγPLIs. Dark grey: consensus match; light grey: one or more amino acid residues do not match the consensus; white: amino acid residue does not match the consensus.
