# Supplementary material for: Identification and characterization of the first endogenous phospholipase A2 inhibitor from a non-venomous tropical snake, Boa constrictor (Serpentes: Boidae)
Source: J Venom Anim Toxins Incl Trop Dis. 2020 Mar 13;26:e20190044. doi: 10.1590/1678-9199-JVATITD-2019-0044 (PMC7092641; doi:10.1590/1678-9199-JVATITD-2019-0044)
Supplement: Additional file 2. [file 1678-9199-jvatitd-26-e20190044-s2.pdf]

Supplementary Material to "Identification and characterization of the first endogenous phospholipase A_2 inhibitor from a non-venomous tropical snake, *Boa constrictor* (Serpentes: Boidae)"

Additional file 2. Gonnet's matrix

Family	Boidae	Colubridae			Pythonidae	Viperidae											Elapidae			
Species	*Bc*	*Ec*	*Eq*	*Sa*	*Mr*	*Bm*	*Bn*	*Bju*	*Bja*	*Be*	*Ba*	*Cdt*	*Lm*	*Gb*	*Pf*	*Pe*	*Ns*	*Om*	*Os*	*Pt*
Bc	**100.0**	66.7	65.6	63.6	58.8	92.8	91.9	96.7	95.0	91.9	96.7	92.3	91.7	89.5	83.4	82.9	60.1	62.8	60.7	63.4
Ec	82.0	**100.0**	98.4	87.0	56.3	65.6	64.2	65.0	66.7	63.1	66.1	66.7	63.4	69.4	67.2	67.2	73.8	76.0	73.2	75.4
Eq	80.3	98.4	**100.0**	86.4	56.8	64.5	63.1	63.9	65.6	62.0	65.0	66.7	62.8	68.3	66.1	66.1	73.8	75.4	72.7	74.9
Sa	77.2	91.8	91.8	**100.0**	54.9	63.6	61.7	62.5	64.1	60.6	63.6	63.6	60.9	65.2	63.6	63.6	69.6	71.7	69.0	72.8
Mr	76.4	74.3	74.9	73.4	**100.0**	58.8	55.9	59.3	58.8	56.5	59.3	58.8	56.6	59.9	54.9	54.4	55.7	54.1	53.6	55.2
Bm	94.5	78.7	77.0	74.5	74.2	**100.0**	88.1	92.8	94.5	88.1	92.8	89.0	87.8	86.7	82.3	82.3	59.6	61.7	59.6	62.8
Bn	94.1	78.6	77.0	73.4	73.7	90.3	**100.0**	91.4	90.3	96.2	92.4	86.5	84.9	85.4	78.4	77.8	57.8	61.0	58.8	61.5
Bju	97.2	79.8	78.1	75.0	75.8	93.9	92.4	**100.0**	93.9	91.4	97.2	91.2	90.1	87.8	81.8	81.2	58.5	61.2	59.0	62.3
Bja	97.8	81.4	79.8	76.6	76.4	94.5	94.1	96.1	**100.0**	90.3	93.9	90.1	89.0	88.4	84.0	83.4	59.6	62.8	60.7	63.4
Be	93.5	79.1	77.5	73.9	73.7	89.7	96.8	91.9	93.5	**100.0**	91.9	86.5	84.9	85.4	78.9	78.4	56.7	59.4	57.2	59.9
Ba	98.3	80.3	78.7	75.5	76.4	94.5	93.5	97.8	97.2	93.0	**100.0**	91.7	90.1	88.4	82.3	81.8	59.0	61.7	59.6	62.8
Cdt	97.2	81.4	80.9	78.3	74.7	91.7	91.4	94.5	95.0	90.8	95.6	**100.0**	91.2	89.0	84.0	83.4	61.2	63.9	61.7	64.5
Lm	91.7	75.4	74.3	72.3	71.4	88.4	85.9	90.1	89.5	85.4	90.6	91.2	**100.0**	85.6	81.2	80.7	58.5	61.2	59.0	62.3
Gb	97.8	82.0	80.3	78.3	74.7	92.8	93.0	95.0	96.7	92.4	96.1	95.6	90.1	**100.0**	86.7	85.1	62.8	67.2	65.0	67.2
Pf	93.9	81.4	79.8	77.7	72.0	88.4	88.1	91.2	91.7	88.1	92.3	92.8	87.3	93.9	**100.0**	97.2	61.2	66.7	64.5	65.6
Pe	93.9	81.4	79.8	77.2	72.5	88.4	88.1	91.2	91.7	88.1	92.3	92.8	87.3	93.9	98.9	**100.0**	62.3	67.2	65.0	66.1
Ns	76.0	84.2	84.2	82.1	70.5	73.2	72.7	73.8	75.4	72.2	74.3	77.0	72.1	76.5	76.5	77.6	**100.0**	88.5	86.3	86.9
Om	76.0	83.6	83.1	82.1	69.9	72.7	73.3	73.8	75.4	72.2	74.3	77.0	71.6	76.5	76.0	77.0	92.9	**100.0**	91.3	92.3
Os	73.2	79.8	79.2	78.8	67.2	69.9	70.6	71.0	72.7	70.1	71.6	74.3	68.9	73.8	73.8	74.9	89.6	91.3	**100.0**	88.0
Pt	76.5	84.2	83.6	82.1	72.1	73.8	73.8	74.9	76.0	73.3	75.4	77.6	73.2	77.6	77.0	78.1	91.3	92.9	88.5	**100.0**

Identity scores (IS)

Similarity scores (SS)

Legend: *Bc - Boa constrictor; Ec - Elaphe climacophora; Eq - Elaphe quadrivirgata; Sa - Sinnonatrix annularis; Mr - Malayopython reticulatus; Bm - Bothrops moojeni; Bn - Bothrops neuwiedi; Bju - Bothrops jararacussu; Bja - Bothrops jararaca; Be - Bothrops erythromelas; Ba - Bothops alternatus; Cdt - Crotalus durissus terrificus; Lm - Lachesis muta; Gb - Gloydius brevicaudus; Pf - Protobothrops flavoviridis; Pe - Protobothrops elegans; Ns - Notechis sutatus; Om - Oxyuranus microlepidotus; Os - Oxyuranus scutellatus; Pt - Pseudonaja textilis.*

Additional file 2 - Gonnet's similarity matrix obtained after multiple sequence alignment of sbγPLI from data bases (see Material and Methods), except for *B. constrictor* (present study). Percentages above and below the 100% diagonal line of the matrix represent identity (left side) and similarity (bottom line) scores, respectively, as indicated. Snake species families are identified on the top line.
